# Alterations of apparent diffusion coefficient from ultra high *b*‐values in the bilateral thalamus and striatum in MRI‐negative drug‐resistant epilepsy

**DOI:** 10.1002/epi4.12990

**Published:** 2024-06-29

**Authors:** Guixian Tang, Hailing Zhou, Chunyuan Zeng, Yuanfang Jiang, Ying Li, Lu Hou, Kai Liao, Zhiqiang Tan, Huanhua Wu, Yongjin Tang, Yong Cheng, Xueying Ling, Qiang Guo, Hao Xu

**Affiliations:** ^1^ Department of Nuclear Medicine, PET/CT‐MRI Center, Center of Cyclotron and PET Radiopharmaceuticals The First Affiliated Hospital of Jinan University Guangzhou China; ^2^ Department of Radiology Central People's Hospital of Zhanjiang Zhanjiang China; ^3^ Epilepsy Center, Guangdong 999 Brain Hospital Affiliated Brain Hospital of Jinan University Guangzhou China

**Keywords:** diffusion, MRI‐negative drug‐resistant epilepsy, striatum, thalamus, ultra high *b*‐values

## Abstract

**Objective:**

Subcortical nuclei such as the thalamus and striatum have been shown to be related to seizure modulation and termination, especially in drug‐resistant epilepsy. Enhance diffusion‐weighted imaging (eDWI) technique and tri‐component model have been used in previous studies to calculate apparent diffusion coefficient from ultra high *b*‐values (ADCuh). This study aimed to explore the alterations of ADCuh in the bilateral thalamus and striatum in MRI‐negative drug‐resistant epilepsy.

**Methods:**

Twenty‐nine patients with MRI‐negative drug‐resistant epilepsy and 18 healthy controls underwent eDWI scan with 15 *b*‐values (0–5000 s/mm^2^). The eDWI parameters including standard ADC (ADCst), pure water diffusion (*D*), and ADCuh were calculated from the 15 *b*‐values. Regions‐of‐interest (ROIs) analyses were conducted in the bilateral thalamus, caudate nucleus, putamen, and globus pallidus. ADCst, *D*, and ADCuh values were compared between the MRI‐negative drug‐resistant epilepsy patients and controls using multivariate generalized linear models. Inter‐rater reliability was assessed using the intra‐class correlation coefficient (ICC) and Bland–Altman (BA) analysis. False discovery rate (FDR) method was applied for multiple comparisons correction.

**Results:**

ADCuh values in the bilateral thalamus, caudate nucleus, putamen, and globus pallidus in MRI‐negative drug‐resistant epilepsy were significantly higher than those in the healthy control subjects (all *p* < 0.05, FDR corrected).

**Significance:**

The alterations of the ADCuh values in the bilateral thalamus and striatum in MRI‐negative drug‐resistant epilepsy might reflect abnormal membrane water permeability in MRI‐negative drug‐resistant epilepsy. ADCuh might be a sensitive measurement for evaluating subcortical nuclei‐related brain damage in epilepsy patients.

**Plain Language Summary:**

This study aimed to explore the alterations of apparent diffusion coefficient calculated from ultra high *b*‐values (ADCuh) in the subcortical nuclei such as the bilateral thalamus and striatum in MRI‐negative drug‐resistant epilepsy. The bilateral thalamus and striatum showed higher ADCuh in epilepsy patients than healthy controls. These findings may add new evidences of subcortical nuclei abnormalities related to water and ion hemostasis in epilepsy patients, which might help to elucidate the underlying epileptic neuropathophysiological mechanisms and facilitate the exploration of therapeutic targets.

**Key points**

- Diffusion‐weighted imaging (DWI) with ultra high *b*‐values may be valuable for the detection of membrane water permeability alterations.
- Apparent diffusion coefficient calculated using ultra high *b*‐values (ADCuh) is higher in the bilateral thalamus and striatum in MRI‐negative drug‐resistant epilepsy compared with healthy controls.
- ADCuh alterations may provide new insight into the neuropathophysiological mechanism of epilepsy.

## 1 INTRODUCTION

Epilepsy is a common and severe neurological disorder characterized by an enduring predisposition to generate epileptic seizures,^1^ which affects over 70 million people worldwide.^2^ Despite the development of new antiepileptic drugs (AEDs) over the past decades, there are still approximately one‐third of patients with epilepsy remain resistant to pharmacotherapy.^3^, ^4^ Patients with drug‐resistant epilepsy have increased risks of premature death, injuries, psychosocial dysfunction, and a reduced quality of life.^5^ However, the underlying neuropathological mechanism of drug‐resistant epilepsy remains largely unknown.

In the past, epilepsy has been considered, for the most part, to be a cortical disease. However, cumulative findings have demonstrated that epileptic seizures involve widespread network interactions between cortical and subcortical structures.^6^, ^7^ Subcortical structures play a crucial role in behavioral manifestations, propagation, and, in some cases, initiation of epileptic seizures,^7^ thus emerging as a critical area to help understanding the pathological mechanism of epilepsy. For example, a longitudinal study using resting‐state functional magnetic resonance imaging (rs‐fMRI) in drug‐resistant epilepsy found abnormal spontaneous brain activity before and after surgery in the deep nuclei including the putamen, pallidum, and the thalamus.^8^ Another rs‐fMRI study also showed dysfunction of the caudates, putamen, and the thalamus in drug‐resistant epilepsy.^9^ A recent magnetic resonance fingerprinting study revealed bilateral tissue‐property changes in the normal‐appearing thalamus and basal ganglia, suggesting subcortical impairment in patients with intractable focal epilepsy.^10^ Changes in activity of striato‐thalamo‐cortical network preceded generalized spike wave discharges,^11^ and both the thalamus and basal ganglia displayed high values of epileptogenicity in focal epilepsy.^12^ In the rat brain, elicited by status epilepticus, regional cerebral blood volume increased in the striatum and thalamus.^13^ In addition, the thalamus and the basal ganglia are potential targets for deep brain stimulation (DBS),^14^, ^15^ which further suggests the crucial role of subcortical nuclei in seizure modulation and termination, especially in drug‐resistant epilepsy.

Diffusion‐weighted magnetic resonance imaging (DWI) is a widely used non‐invasive technique that is sensitive to the random motion of water molecules in biological tissues, and offers information about tissue architecture and pathological changes on a cellular level.^16^ The commonly used quantitative parameter to interpret DWI is apparent diffusion coefficient (ADC) value.^17^ ADC value is often estimated by fitting the signal intensities (SIs) from a series of DWI with different diffusion weightings (*b* values) using a mono‐exponential model.^18^, ^19^ Intravoxel incoherent motion imaging (IVIM), a bi‐exponential model of DWI, considers the combined effects of pure molecular diffusion (*D*) and pseudo‐diffusion or blood perfusion (D*).^20^ This model requires multiple *b*‐values, including low *b*‐values (<200 s/mm^2^) for measuring blood perfusion (fast diffusion component) and high *b*‐values (usually range from 200 to 1000 s/mm^2^) for measuring true water diffusion (slow diffusion component).^20^, ^21^, ^22^, ^23^ However, this bi‐component model does not consider the exchange effects between the fast and slow diffusion components and membrane permeability.^24^ To date, researchers have used enhance diffusion‐weighted imaging (eDWI) technique and tri‐component model to calculate standard ADC (ADCst), ADCuh, D*, and *D* in diseases such as Parkinson's disease,^25^ prostate cancer,^26^ and bipolar disorder.^27^ The tri‐component model suggest that with higher b values (eg, *b* > 1500 s/mm^2^), the diffusion MRI could reflect the non‐Gaussian nature in tissues (hindrance of diffusion by tissue elements, such as cell membranes).^24^ The discovery of aquaporin water channel proteins has provided insight into the molecular mechanism of membrane water permeability,^28^ which is critical for extracellular solute concentrations and neuronal excitability.^29^ Notably, evidences from animal and human histopathological studies have shown the correlation between ADC values and aquaporins (AQPs) expression levels.^30^, ^31^, ^32^, ^33^ There is growing evidences about the role of AQPs play in the pathogenesis of epilepsy, such as ions homeostasis mechanism.^34^ We therefore hypothesized that eDWI technique is useful in exploring potential microenvironment change in the subcortical nuclei of drug‐resistant epilepsy in vivo.

Therefore, in the current study, we aimed to investigate the possible alterations of ADCuh in the thalamus and the striatum in MRI‐negative drug‐resistant epilepsy by using eDWI. To the best of our knowledge, this is the first study to reveal the changes of ADCuh in the thalamus and the striatum in MRI‐negative drug‐resistant epilepsy, which might indirectly reflect membrane water permeability alterations of these regions and help to elucidate the neuropathophysiological mechanism of the disease.

## 2 METHODS

### 2.1 Study population

Twenty‐nine patients with MRI‐negative drug‐resistant epilepsy underwent MRI scan at the PET‐CT/MRI center of the First Affiliated Hospital of Jinan University between January 1, 2014 and June 30, 2020. Patients were recruited from the epilepsy center, Guangdong 999 Brain Hospital (Affiliated Brain Hospital of Jinan University), Guangzhou, China. Inclusion criteria for epilepsy patients were as follows: (1) patients diagnosed with drug‐resistant epilepsy according to the standards of the International League Against Epilepsy (ILAE); (2) no structural lesions causing seizures were found at 3.0 T MRI; (3) age between 18 to 55 years; (4) complete clinical data of patients.

Eighteen age‐ and gender‐matched healthy controls (HCs) were recruited through local advertisements. Exclusion criteria were: the presence of any neurological or psychiatric illness, the presence of a family history of neurological or psychiatric illness, and any history/current significant medical illness.

The study was approved by the Ethics Committee of First Affiliated Hospital of Jinan University (China), and the methods and procedures were carried out in accordance with the approved guidelines. All subjects signed a written informed consent form after a full written and verbal explanation of the study.

### 2.2 MRI protocol

All MRI data were obtained from a 3.0 Tesla MR scanner (Discovery MR 750 System, GE Healthcare, Milwaukee, WI) with an 8‐channel phased array head coil. Subjects were scanned in a supine, head‐first position. Head cushions were placed on both sides of the head symmetrically to constrain head movements of the subjects.

The eDWI data were acquired using Spin echo‐echo planar imaging (SE‐EPI) sequence with 15 different *b* values (0, 30, 50, 100, 200, 300, 500, 800, 1000, 1, 500, 2000, 3000, 3500, 4000, and 5000 s/mm^2^). Other imaging parameters were: TR = 3000 ms, TE = 96 ms, NEX = 1, number of slices = 24, bandwidth = 250 kHz, slice thickness/gap = 3.0/0.3 mm, field of view (FOV) = 240 × 240 mm, matrix = 128 × 128, and voxel resolution = 0.938 × 0.938 × 3.0 mm. Eddy current correction was applied with real time field adjustment and real‐time correction of motion‐induced phase error to obtain high‐quality diffusion images. Spatial coverage for eDWI was from the lower margin of the pons to the upper margin of basal ganglia. The total scan time was 6 min 51 s. In addition, a three‐dimensional (3D) brain volume imaging (3D BRAVO) sequence covering the whole brain was used for structural data acquisition with: TR/TE = 8.2/3.2 ms, slice thickness/gap = 1.0/ 0 mm, matrix = 256 × 256, FOV = 240 × 240 mm, NEX = 1, flip angle = 12°, bandwidth = 31.25 Hz, and acquisition time = 3 min 45 s. Other sequences included 3D fast spin‐echo (FSE) T2‐weighted imaging (T2 WI), and 3D Cube T2 fluid attenuation inversion recovery (T2 FLAIR). MRI was classified as negative when no structural lesions causing seizures were detected.^35^

### 2.3 MR data processing

The data were transferred to a dedicated workstation (General Electric Advantage Workstation 4.5) where the eDWI data were post‐processed using Functool software version 9.4.05a. ADCst, *D*, and ADCuh maps were calculated from eDWI sequence by fitting all diffusion weighted images and the b0 image into a tri‐component model^25^: where the diffusion weighted signal *S* is fit to the bi‐exponential equation when *b* values are less than 2000 s/mm^2^, and the mono‐exponential equation is used to quantify the ADCuh with *b*‐values ≧2000 s/mm^2^. ADCst is the standard ADC, *f* is the perfusion fraction, *D* is the pure diffusion coefficient that reflects the random motion of water molecules, and D* is the pseudo‐diffusion coefficient that reflects fast, or perfusion based, molecular diffusion. ADCuh is the apparent diffusion coefficient calculated by fitting the five ultra high *b*‐values (2000, 3000, 3500, 4000, and 5000 s/mm^2^) to the mono‐exponential equation. The algorithms were implemented in the workstation (General Electric Advantage Workstation 4.5), which allowed the extraction of parametric maps representing ADCst, *D*, and ADCuh on a pixel‐by‐pixel basis.

**FIGURE 1:**
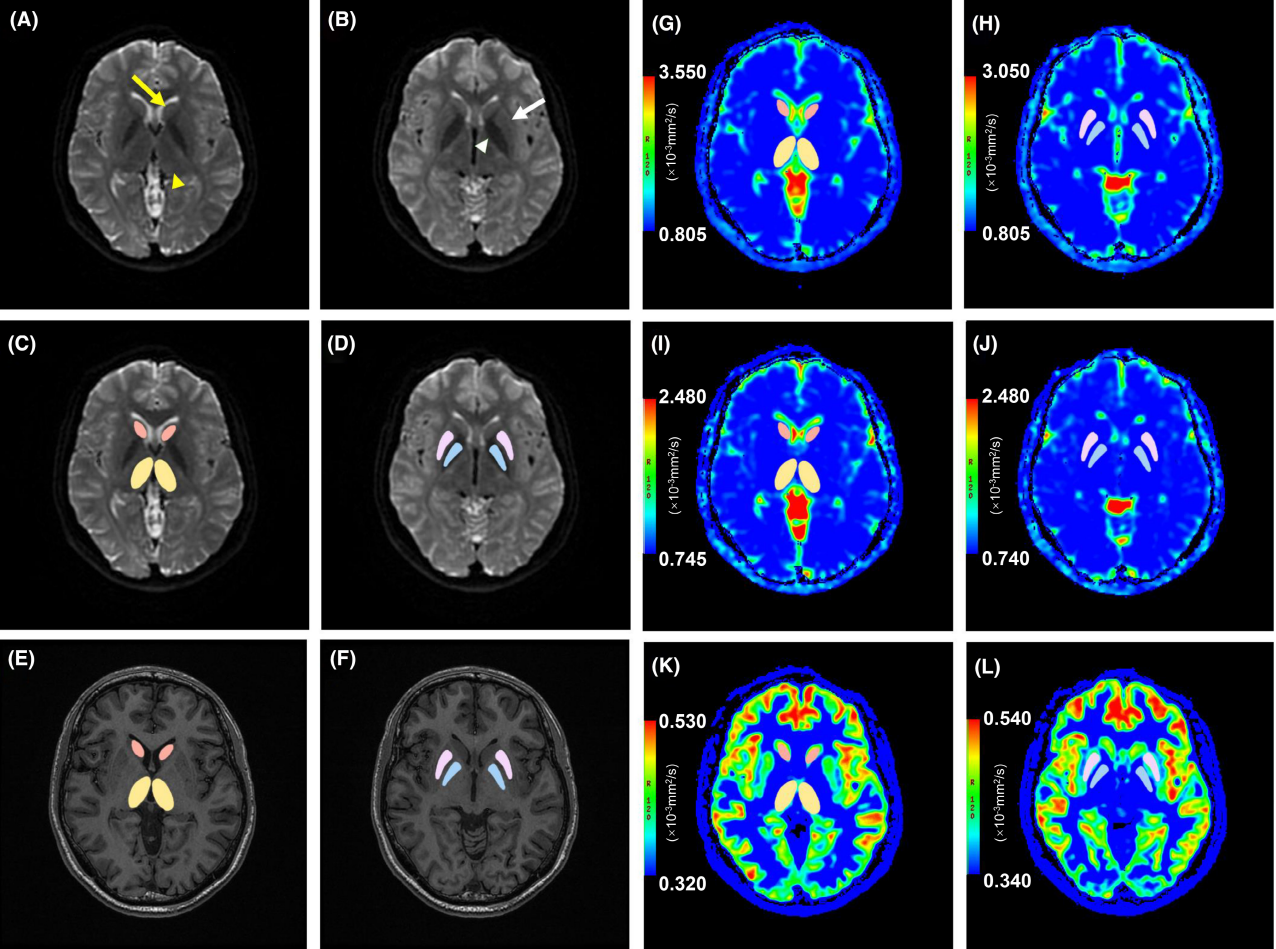
Procedure used to draw region of interests (ROIs) in the bilateral thalamus, caudate nucleus, putamen, and globus pallidus on the maximum level of the ROIs structures.

Eight separate regions of interest (ROIs) were manually placed in the bilateral thalamus, caudate nucleus, putamen, and globus pallidus on the maximum level of the ROIs structures on b = 0 images (which is essentially a T2‐weighted image). All ROIs were determined by two independent neuroradiologists (T.G and T.Y, with 5 and 10 years of experience, respectively) at the workstation. Considering the relatively low resolution of the eDWI, we used high‐resolution 3D BRAVO images as reference to better distinguish the ROIs from the surrounding structures. For each subject, ROI sizes were identical in the left and right by using the mirror symmetry tools from the Functool software. The b0 image for the slices with the bilateral thalamus, caudate nucleus, putamen, and globus pallidus (Figure 1A–D) and the high‐resolution 3D BRAVO images (Figure 1E,F) illustrate how and where the ROIs were drawn in these areas. Major vascular structures, cerebral spinal fluid, and artifacts were avoided in placing the ROIs. All the ROIs were then transferred to the maps of ADCst (Figure 1G,H), *D* (Figure 1I,J), and ADCuh (Figure 1K,L) for measurement. To combine the left and right ROIs into a single measure for each region, the average ADCst, *D*, and ADCuh values of left and right hemisphere for each region were calculated for statistical analyses.

### 2.4 Statistical analyses

Statistical analyses were performed using the commercial software (IBM Statistical Package for the Social Sciences 25.0, IBM Corp., Armonk, NY, United States). Distributions of age between the two groups was compared with independent two sample *t*‐test. Chi‐square test was used to compare gender distribution. Inter‐rater reliability was assessed using the intra‐class correlation coefficient (ICC) and Bland–Altman (BA) analysis. Multivariate generalized linear models (GLMs) were performed to compare the eDWI parameters of all the ROIs between epilepsy and HCs groups. Specifically, a separate multivariate generalized linear model (GLM) was used for each diffusion metric comparison (ADCst, *D*, and ADCuh, respectively), with all the ROIs as dependent variables, and age and gender as covariates. As multiple comparisons were conducted, false discovery rate (FDR) correction through Benjamini–Hochberg procedures^36^, ^37^ was applied to control for all the *p* values calculated from GLMs. This statistical analysis process was based on the R Project for Statistical Programming (version 3.4.3; https://www.r‐project.org/). Then, diffusion metric with *p* < 0.05 after FDR correction was seen as significant between epilepsy patients and HCs.

## 3 RESULTS

Demographic and clinical data from epilepsy patients and control subjects are shown in Table 1. There was no significant difference in age (*p* = 0.121) or gender (*p* = 0.512) between epilepsy patients and control subjects.

**TABLE 1:** Demographic and clinical data of the participants.

	EP (*n* = 29)	HC (*n* = 18)	Statistic	
Gender(male/female)	16/13	8/10	*χ* ^*2*^ = 0.474	*p* = 0.512
Age, mean (SD)	22.03 (4.989)	24.11 (3.104)	*t* = −1.582	*p* = 0.121
Age at epilepsy onset, years (IQR)	11.07 (6.5–16.5)			
Duration of epilepsy, years (IQR)	10.55 (6.5–14)			
Presence of FC	4 (13.8%)			
Tonic–clonic seizures	10 (34.5%)			
Early brain injury	4 (13.8%)			
Family history	0 (0%)			
Cerebral anoxia	1 (3.4%)			
CNS infection	2 (6.9%)			
Head trauma	9 (31.0%)			
Previous surgical history	0 (0%)			
Auras	17 (58.6%)			

Abbreviations: %, percent; CNS, central nervous system; EP, MRI‐negative refractory epilepsy patients; FC, febrile convulsion; HC, healthy controls; IQR, inter‐quartile range; n, number; SD, standard deviation.

ADCst, *D*, and ADCuh showed a significantly high ICC value close to 1 (*p* < 0.05; Table 2). BA analysis also showed a small range of 95% limits of agreement, with the difference near 0. Hence, all measurements were regarded as reliable among different raters.

**TABLE 2:** Inter‐rater reliability and Bland–Altman analysis results of diffusion parameters in the bilateral thalamus, caudate nucleus, putamen, and globus pallidus.

Region	Parameter (×10^−3^ mm^2^/s)	Side	ICC	*p*	Difference	95% LOA
Thalamus	ADCst	L	0.748	<0.001	0.0005	(−0.059, 0.06)
		R	0.647	<0.001	−0.003	(−0.09, 0.084)
	D	L	0.942	<0.001	0.0009	(−0.03, 0.031)
		R	0.825	<0.001	−0.0043	(−0.051, 0.042)
	ADCuh	L	0.715	<0.001	−0.0003	(−0.029, 0.028)
		R	0.919	<0.001	−0.0016	(−0.022, 0.019)
Caudate nucleus	ADCst	L	0.874	<0.001	−0.0016	(−0.058, 0.055)
		R	0.795	<0.001	0.007	(−0.067, 0.081)
	D	L	0.951	<0.001	−0.0002	(−0.04, 0.04)
		R	0.845	<0.001	0.0064	(−0.044, 0.057)
	ADCuh	L	0.934	<0.001	−0.0006	(−0.018, 0.017)
		R	0.959	<0.001	0.0016	(−0.014, 0.017)
Putamen	ADCst	L	0.778	<0.001	−0.0005	(−0.037, 0.036)
		R	0.716	<0.001	−0.0099	(−0.054, 0.034)
	D	L	0.855	<0.001	−0.0014	(−0.052, 0.05)
		R	0.656	<0.001	−0.0089	(−0.068, 0.05)
	ADCuh	L	0.881	<0.001	0.0085	(−0.021, 0.038)
		R	0.843	<0.001	0.0105	(−0.024, 0.045)
Globus pallidus	ADCst	L	0.739	<0.001	0.0003	(−0.099, 0.1)
		R	0.707	<0.001	−0.0113	(−0.125, 0.102)
	D	L	0.685	<0.001	0.0016	(−0.16, 0.164)
		R	0.616	0.001	−0.0099	(−0.207, 0.187)
	ADCuh	L	0.821	<0.001	−0.0121	(−0.07, 0.046)
		R	0.862	<0.001	−0.0023	(−0.056, 0.051)

Abbreviations: ADCst, ADC calculated using the standard *b*‐values; ADCuh, ADC calculated using the ultra high *b*‐values; *D*, pure water diffusion coefficient; ICC, intraclass correlation; L, left; LOA, limits of agreement; R, right.

**FIGURE 2:**
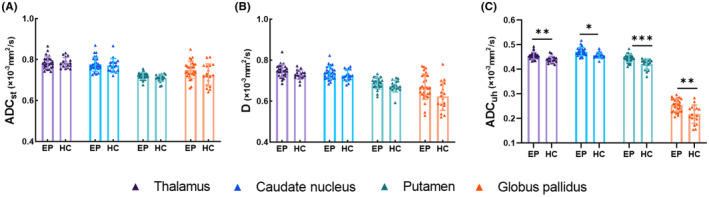
Group differences of the parameters generated from enhance diffusion‐weighted imaging (eDWI).

Table 3 showed the group differences of the eDWI parameters between EP and HCs. After FDR correction, the epilepsy group showed increased ADCuh values in the bilateral thalamus, caudate nucleus, putamen, and globus pallidus compared with HCs (all FDR‐*p* < 0.05; Figure 2). No significant difference was observed in the remaining eDWI parameters of these ROIs between the two groups (all FDR‐*p* > 0.05).

**TABLE 3:** General linear model statistical analyses of the average ADCst, *D*, and ADCuh values of the bilateral thalamus, caudate nucleus, putamen, and globus pallidus between patients with MRI‐negative epilepsy and HC subjects.

Region	Parameter (×10^−3^ mm^2^/s)	EP	HC	*F* values	*p* values	FDR‐*p* values
		(*n* = 29)	(*n* = 18)			
Thalamus	ADCst	0.785 ± 0.03	0.785 ± 0.02	0.075	0.785	0.785
	D	0.748 ± 0.03	0.729 ± 0.02	3.146	0.083	0.166
	ADCuh	0.455 ± 0.01	0.439 ± 0.01	11.775	0.001	0.004**
Caudate nucleus	ADCst	0.776 ± 0.03	0.771 ± 0.03	0.089	0.767	0.785
	D	0.738 ± 0.03	0.725 ± 0.03	1.002	0.322	0.386
	ADCuh	0.471 ± 0.02	0.456 ± 0.01	8.288	0.006	0.018*
Putamen	ADCst	0.720 ± 0.02	0.710 ± 0.02	1.497	0.228	0.341
	D	0.688 ± 0.03	0.673 ± 0.03	1.327	0.256	0.341
	ADCuh	0.444 ± 0.02	0.420 ± 0.02	14.738	<0.001	<0.001***
Globus pallidus	ADCst	0.749 ± 0.04	0.726 ± 0.05	2.279	0.138	0.237
	D	0.671 ± 0.06	0.624 ± 0.07	4.269	0.045	30.108
	ADCuh	0.254 ± 0.03	0.217 ± 0.04	12.858	0.001	0.004**

*Note*: eDWI parameters are reported as mean ± SD in each brain region of interests. General linear model statistical analyses with significant *p*‐values after FDR correction were marked with *.

Abbreviations: ADCst, standard apparent diffusion coefficient; ADCuh, ultra high *b*‐values apparent diffusion coefficient; *D*, pure diffusion coefficients; eDWI, enhance diffusion‐weighted imaging; EP, MRI‐negative epilepsy; FDR, false discovery rate; HC, healthy controls.

* *p* values < 0.05.

** *p* values < 0.01.

*** *p* values < 0.001.

## 4 DISCUSSION

In the current study, we measured the eDWI parameters in MRI‐negative drug‐resistant epilepsy patients. Compared to HCs, the MRI‐negative drug‐resistant epilepsy patients showed significantly increased ADCuh in the bilateral thalamus and striatum, indicating increased transmembrane water exchange in these regions.

The thalamus is a densely connected collection of nuclei that plays a critical role in ongoing cortical functioning.^38^, ^39^ As a major node in the epilepsy networks,^40^ this region is very important in the initiation, propagation, and inhibition of epileptic activity.^41^, ^42^, ^43^ Previous histopathological data illustrated swelling of dendrites in the thalamus and dark cell degeneration in both the cortex and thalamus reflect a seizure‐induced, excitotoxic process mediated by excessive release of glutamate at synaptic receptors.^44^ Animal studies suggested that neuronal necrosis in neocortex and thalamic nuclei could be caused by status epilepticus in well‐oxygenated rats,^45^ and MRI changes in the rat brain were correlated with tissue and cell damage in the medial thalamus elicited by status epilepticus.^13^ A number of studies in drug‐resistant epilepsy have reported the alterations in morphometry,^46^, ^47^ functional activity,^8^ and functional connectivity^46^ in the thalamus. A multimodal magnetic resonance encephalography study indicated electrophysiological and hemodynamic changes in the thalamus in drug‐resistant epilepsy.^48^ Using diffusion tensor imaging (DTI), altered microstructure of thalamo‐cortical fibers were more obvious in drug‐resistant epilepsy compared with non‐drug‐resistant epilepsy.^49^ Another DTI study indicated widespread bilateral structural connectivity abnormalities in the thalamocortical pathways in drug‐resistant epilepsy.^50^ In MRI‐negative newly diagnosed focal epilepsy patients, connectomes with increased diffusivity (edge between right thalamus and left parietal nodes) were found in patients with persistent seizures compared to patients who became seizure‐free, suggesting altered structural connectome in the thalamus were related to pharmaco‐resistance.^51^ Also, stimulation of the thalamus has been shown to decrease the frequency of drug‐resistant seizures.^52^, ^53^, ^54^, ^55^ Overall, the above evidence might support our findings of alterations of ADCuh in the thalamus, suggesting that the thalamus may play a critical role in the neuropathophysiological mechanism of drug‐resistant epilepsy.

Another crucial finding in this study was the significantly increased ADCuh in the striatum, including bilateral caudate nucleus, putamen, and the globus pallidum, in drug‐resistant epilepsy compared with HCs. The striatum could induce an overall inhibitory effect on the thalamus and exert overall excitatory effects on the cortex,^56^ thus playing an important role in maintaining the activity of epilepsy.^57^ It is reported that impaired cortico‐striatal excitatory transmission could trigger epilepsy in mice.^58^ Also, drug resistance was strongly associated with the overexpression of P‐glycoprotein observed in the striatum in drug‐resistant seizure model in mice.^59^ In an intractable epilepsy case, transient seizure disappeared due to bilateral striatal necrosis, which suggests that the striatum may be involved in the propagation pathway for epileptic seizure activity.^60^ A previous single photon emission computed tomography (SPECT) study showed the putamen had abnormal perfusion patterns during interictal and ictal in focal drug‐resistant epilepsy.^61^ Using positron emission tomography (PET), researchers have observed a reduction of striatal dopamine uptake in drug‐resistant epilepsy.^62^, ^63^, ^64^ Rs‐fMRI studies showed drug‐resistant epilepsy exhibited altered topological properties in the caudate nucleus,^65^, ^66^ and temporal lobe drug‐resistant epilepsy was associated with volume loss in the striatal regions including the caudate and pallidum.^62^, ^67^ In patients with MRI‐negative cortical epilepsy, structural and DTI studies have revealed smaller volumes, microstructural anomalies, and abnormal network characteristics in subcortical regions including the putamen, caudate nucleus, and the globus pallidus.^68^, ^69^ Taken together, these evidences suggested the abnormities of the striatum in drug‐resistant epilepsy. Moreover, being consistent with previous findings,^25^, ^27^ in this study, the ADCuh values in both epilepsy and controls are lower than ADCst and *D* values, which may be explained by diffusion signal decay of the brain tissue with the increase of *b*‐values.^70^

The AQPs are a group of specific transmembrane channel proteins responsible for water molecules crossing cell membranes, among which the AQP1, AQP4, and AQP9 have been demonstrated to distribute in the brain tissue.^71^ The AQPs are important factors in water and potassium homeostasis.^72^ The AQP4 and AQP9 both express in astrocytes, and AQP1 express in the choroid plexus; the expression of AQP1 can be regulated by ubiquitination, and that osmolality can regulate the expression of AQP1, AQP4, and AQP9.^73^ The correlations between ADCuh and AQPs have been demonstrated in previous studies. In ultra high *b*‐values studies of kidney disease, ADCuh values were significantly higher in the kidneys, and were positively correlated with AQP2 expression in a rat model of diabetic nephropathy^74^; however, in that study, AQP4 expression was unchanged. Similarly, another study reported ADCuh was positively correlated with AQP1 and AQP2 expression in a rabbit model of renal artery stenosis.^31^ Besides, the ultra high *b*‐values studies of brain showed ADCuh was significantly higher in the high‐grade gliomas than in the low‐grade gliomas,^33^, ^75^ which were positively correlated with AQP1^33^, ^75^ and AQP4.^75^ The above divergent correlations between ADCuh with different subtypes of AQPs might be explained as each AQP subtype displaying a different pattern of localization and expression.^76^ Based upon these findings, we propose that ADCuh might be able to assess water transportation by AQPs. In the previous ultra high *b*‐values study of Parkinson's disease,^25^ the ADCuh of the pallidum and putamen in the patient group were significantly lower than that of the corresponding region in the control group, which reflects the decrease of water transportation across membrane. This might be related to astrocyte senescence in Parkinson's disease as a neurodegenerative disorder.^77^ And widespread loss of perivascular AQP4 polarization along the penetrating arteries accompanied the decline in cerebrospinal fluid‐interstitial fluid have been reported in aging mice.^78^ On the contrast, we found increased ADCuh values in the striatum in MRI‐negative drug‐resistant epilepsy patients in the current study, which might suggest increased transport of water across membranes by AQPs. Previous studies have reported increased AQP1 expression of astrocytes in surgical samples of the anterior temporal neocortex of patients with drug‐resistant epilepsy.^79^ And in animal model, AQP4‐overexpressing mice had an accelerated progression of cytotoxic brain swelling.^80^ Astrocyte swelling above the baseline state is directly tied to neuronal excitability increases, and in the extreme can push tissue excitability into a pathological state, triggering seizures.^81^ Taken together, our results indirectly revealed disrupted ion and water homeostasis in the thalamus and striatum in MRI‐negative drug‐resistant epilepsy, providing clues to the underlying pathophysiology of epileptogenic properties in the subcortical nuclei.

There are several limitations of this study. First, the patient population was relatively small, and future studies should include larger populations of subjects to verify our findings. Second, the present study only included samples of MRI‐negative drug‐resistant epilepsy which might not only avoid the potential confounds of the lesion differences, but also limited the generalizability of our results to other type of epilepsy such as MRI‐positive drug‐resistant epilepsy and non‐drug‐resistant epilepsy. Future studies should explore the eDWI parameters in other types of epilepsy. Third, the present study was based on priori hypothesis rather than data‐driven research, so only the eDWI parameters of the thalamus and striatum were explored, which might result in a selection bias. Future research is acquired to investigate ADCuh values in other brain areas in epilepsy. Fourth, the current results could not be confirmed by pathology due to the biopsy tissue of the thalamus and striatum was not available from epilepsy patients according to the ethical regulations, however, previous studies about AQPs expression profiles from experiments of rodents^82^, ^83^ may partly support the alteration of the ADCuh values in this study. Fifth, ADCuh values might relate to the time between the MRI acquisitions and the most recent seizure of patients. Future study should take this into consideration. Finally, higher *b*‐values might lead to a decreased signal‐to‐noise ratio. However, the image of higher *b*‐values is of sufficient quality to recognize the ROIs of the thalamus and striatum, and high‐resolution 3D BRAVO images were combined with b0 images to improve the accuracy of measurement.

In conclusion, our preliminary study demonstrated the abnormalities of the ADCuh values in the bilateral thalamus and striatum in MRI‐negative drug‐resistant epilepsy patients using eDWI technique. The results depicted that MRI‐negative drug‐resistant epilepsy patients exhibited higher ADCuh values in the bilateral thalamus and striatum compared with controls, which might reflect membrane water permeability alterations in these regions. These findings may provide a novel insight to the subcortical nuclei‐related neuropathophysiological mechanisms underlying drug‐resistant epilepsy.

## FUNDING INFORMATION

This work was financially supported by the National Natural Science Foundation of China (No. 81871383), Basic and Applied Basic Research Foundation of Guangdong (No. 2020A1515011192), Guangzhou Science and Technology Program, China (No. 2023A03J0610), and Frontier Technology Program of the Affiliated of Jinan University, China (No. JNU1AF‐CFTP‐2022‐n1214).

## CONFLICT OF INTEREST STATEMENT

The authors of this manuscript declare no relationships with any companies, whose products or services may be related to the subject matter of the article. We confirm that we have read the Journal's position on issues involved in ethical publication and affirm that this report is consistent with those guidelines.

## ETHICS STATEMENT

The studies involving human participants were reviewed and approved by the First Affiliated Hospital of Jinan University Research Ethics Committee.

## INFORMED CONSENT

Written informed consent was obtained from the individuals for the publication of any potentially identifiable images or data included in this article.

## STATISTICS AND BIOMETRY

No complex statistical methods were necessary for this paper.

## STUDY SUBJECTS OR COHORTS OVERLAP

No study subjects or cohorts have been previously reported.

## METHODOLOGY

Methodology:

- Retrospective
- Case–control study
- Performed at one institution

## GUARANTOR

The scientific guarantor of this publication is Hao Xu.
